# Journal of the International AIDS Society: an important step forward

**DOI:** 10.1186/1758-2652-11-1

**Published:** 2008-09-22

**Authors:** Mark A Wainberg, Elly Katabira, Shirin Heidari

**Affiliations:** 1 McGill University AIDS Centre, Jewish General Hospital, Montreal, Quebec, Canada; 2 Makerere Medical School, Kampala, Uganda; 3 International AIDS Society, Geneva, Switzerland

## Abstract

This editorial welcomes readers to the launch of Journal of the International AIDS society.

## Editorial

*Journal of the International AIDS Society *(*JIAS*) was re-launched at the International AIDS Conference in Mexico in August 2008.

After four years of fruitful collaboration with *Medscape Journal of Medicine*, which allowed *JIAS *to operate and grow under its umbrella, we have reached the time when we are able to become an independent journal and continue with this success. Our gratitude must go to our former partner Medscape for this collaboration, which we now hope to continue in new forms.

*Journal of the International AIDS Society *(*JIAS*) is now proud to be associated with BioMed Central as its new publisher.

BioMed Central has a long history of successful publications in a wide array of disciplines in medical science. They are professional and have attained high levels of success by providing an artful combination of rapid peer review, open access, and a professional staff that strives at all times to help journals to sustain high quality publications.

The achievements of BioMed Central in the field of HIV/AIDS in recent years is perhaps best illustrated by the success of two sister journals; *Retrovirology *and *AIDS Research and Therapy*, journals with which *JIAS *is committed to work with closely in the future.

An important key to the success of medical journals is the ability to engage serious and well-respected reviewers who are willing to donate their time to assist in the peer review process. This is a formula for success that *JIAS *will now adopt to attain the goals that we, the Editors-in-Chief and our sponsor, the International AIDS Society, have set.

As in the past, we will give priority to articles that are submitted by authors in developing countries and will insist that all articles accepted for publication be of high quality. In order to maintain a high standard of publication, *JIAS *is committed to provide mentorship to authors as a means of helping them improve the quality of submitted manuscripts.

We hope that we will receive articles on the all important topic of HIV clinical trials, which now are, to an increasing extent, being carried out in developing country settings. This is a reflection of where the great burden of HIV infection in the world actually is, as well as the fact that an insufficient number of patients available for enrollment into large scale clinical trials currently reside in developed countries. Many new drugs, that are now being tested for the first time in either naive or experienced populations, are increasingly being studied in developing country settings. We hope our journal will create a platform for dissemination of the results of these trials during the coming years.

Of course we also hope to attract excellent manuscripts in areas of research that may be related tangentially to clinical trials. As an example, the study of the prevalence of drug resistance mutations in different countries is a topic of widespread interest as is the extent to which sexual transmission of mutations associated with drug resistance might take place. The occurrence and transmission of such mutations is, of course, an unfortunate yet inevitable consequence of the increasing availability of antiretroviral drugs (ARVs). Frequently, different patterns of such transmissions may occur in different countries based on patterns of drug use. It will be important to document such differences. This is but one example of research that will be encouraged by *JIAS *for potential publication.

The issue of drug resistance clearly has important overlaps with the epidemiology of HIV disease in both developed and developing country settings. Every effort will be made to encourage submissions of manuscripts from epidemiologists who are anxious to describe the state of the HIV epidemic in a variety of developing and developed country settings.

We welcome commentary pieces on important topics. These may include such subjects as government policies in regard to access to antiretroviral drugs and whether or not appropriate efforts are being made to promote use or not of generic products. We will also welcome articles in the social sciences and examples of topics that might be covered include attitudes towards prevention trial research in the aftermath of the failures of phase 3 clinical trials involving potential vaccines and microbicides.

Commentary articles will also be welcome on the issue of government policy toward encouragement of safe sex practices, as will articles on the ethics of various research approaches involving the participation of human subjects in both treatment and prevention trials from both a planning and implementation perspective. We would also like to generate activity on topics that have been relatively neglected and that should be prioritized for future research, including the very important subject of harm reduction and how to persuade policy makers that implementation of certain strategies, that might not be perceived as having popular support, could, in fact, result in diminished rates of HIV transmission.

We will also solicit articles from major funding agencies and key opinion leaders in various fields in regard to the priorities that they might wish to set for research in developing country settings. As an example, invited articles will be solicited from leaders at the National Institute of Health, the Gates Foundation, the International Partnership on Microbicides, and the International AIDS Vaccine Initiative, among many others.

*Journal of the International AIDS Society *(*JIAS*) will closely collaborate with other journals such as *AIDS, Retrovirology *and *AIDS Research and Therapy *to serve the scientific community by disseminating a wide range of scientific findings in the field. In the area of basic science research, we hope to collaborate closely with our sister journal *Retrovirology*. And it is conceivable that we may also decide, on occasion, that a different sister journal may be more appropriate for a manuscript submitted to *JIAS*. We will remain as flexible as possible with decisions of this type.

Our goal is to transform *JIAS *into a highly rated and well respected journal in which to publish high quality manuscripts. In order to realize this goal, we recognize that we will have to work hard. At the same time, we will need to be tough and only accept high quality manuscripts. This is a necessary measure if we are to succeed in the realization of our goals for *JIAS *and the promotion of high quality research in HIV/AIDS from the world at large and developing countries in particular.

*Journal of the International AIDS Society *(*JIAS*) welcomes submissions. We ask colleagues to submit via the online submission tool. We look forward to reviewing and publishing exciting new data and research that advance the struggle against the HIV pandemic.

